# Bridging-induced phase separation induced by cohesin SMC protein complexes

**DOI:** 10.1126/sciadv.abe5905

**Published:** 2021-02-10

**Authors:** Je-Kyung Ryu, Céline Bouchoux, Hon Wing Liu, Eugene Kim, Masashi Minamino, Ralph de Groot, Allard J. Katan, Andrea Bonato, Davide Marenduzzo, Davide Michieletto, Frank Uhlmann, Cees Dekker

**Affiliations:** 1 Department of Bionanoscience, Kavli Institute of Nanoscience Delft, Delft University of Technology, Delft, Netherlands.; 2 Chromosome Segregation Laboratory, The Francis Crick Institute, London, UK.; 3 SUPA, School of Physics and Astronomy, University of Edinburgh, Edinburgh EH9 3FD, UK.; 4 MRC Human Genetics Unit, Institute of Genetics and Molecular Medicine, University of Edinburgh, Edinburgh EH4 2XU, UK.

## Abstract

Structural maintenance of chromosome (SMC) protein complexes are able to extrude DNA loops. While loop extrusion constitutes a fundamental building block of chromosomes, other factors may be equally important. Here, we show that yeast cohesin exhibits pronounced clustering on DNA, with all the hallmarks of biomolecular condensation. DNA-cohesin clusters exhibit liquid-like behavior, showing fusion of clusters, rapid fluorescence recovery after photobleaching and exchange of cohesin with the environment. Strikingly, the in vitro clustering is DNA length dependent, as cohesin forms clusters only on DNA exceeding 3 kilo–base pairs. We discuss how bridging-induced phase separation, a previously unobserved type of biological condensation, can explain the DNA-cohesin clustering through DNA-cohesin-DNA bridges. We confirm that, in yeast cells in vivo, a fraction of cohesin associates with chromatin in a manner consistent with bridging-induced phase separation. Biomolecular condensation by SMC proteins constitutes a new basic principle by which SMC complexes direct genome organization.

## Teaser

Yeast cohesin complexes cluster onto long DNA into liquid droplets, which they accomplish by ATP-independent DNA bridging.

## INTRODUCTION

Members of the structural maintenance of chromosome (SMC) protein family such as condensin, cohesin, and the Smc5/6 complex are key proteins for the spatial and temporal organization of chromosomes (*1*–*4*). Recent in vitro experiments visualized real-time DNA loop extrusion mediated by condensin and cohesin (*5*–*8*). While loop extrusion by SMC proteins constitutes a fundamental building block in the organization of chromosomes, other factors may also contribute. In the past decade, it has become abundantly clear that phase separation plays a role in many processes in biological cells (*9*), including chromosome organization (*10*–*12*). Thus far, SMC proteins have not been implied in this biomolecular condensation. While adenosine 5′-triphosphate (ATP)–independent clustering of DNA and SMC proteins has been reported (*13*–*17*), these observations were often attributed to potentially imperfect protein purification or nonphysiological buffer conditions. For example, Davidson *et al.* (*7*) reported in vitro DNA loop extrusion by the human cohesin complex when the cohesin concentration was limited to very low values (<0.8 nM, i.e., much lower than physiological concentrations of ~333 nM) (*18*, *19*) and mentioned that the cohesin complexes were prone to aggregation at higher concentrations. These findings raise the question whether this aggregate formation may be intrinsic and have a physiological meaning.

Here, we report that interactions between the yeast cohesin SMC complex and DNA lead to pronounced clustering, which is due to a new type of phase separation. We note that the term “phase separation” has become the shorthand nomenclature for a variety of phenomena ranging from protein aggregation to RNA-protein clustering due to liquid-liquid phase separation (LLPS) (*9*). More generally known as “biomolecular condensation,” it describes the spontaneous demixing of biomolecules into a low-density bulk fraction and locally concentrated clusters, which is a reversible equilibrium phenomenon (*20*). The cohesin-DNA clustering behavior that we observe is ATP independent but, unexpectedly, depends on DNA length. We find that single cohesin complexes are able to bridge distant points along DNA that act as nucleation points for recruiting further cohesin complexes—a behavior indicative of bridging-induced phase separation (BIPS) (*21*, *22*), also known as polymer-polymer phase separation (*23*), a type of biomolecular condensation that was studied theoretically but lacked any experimental verification in biological examples so far.

## RESULTS

### Cohesin induces cohesin-DNA cluster formation in an ATP-independent manner

**Fig. 1:**
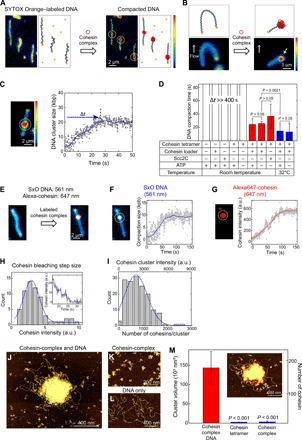
ATP-independent DNA compaction mediated by cohesin holocomplex. (**A**) Snapshots before and after cohesin-induced compaction of a doubly tethered DNA molecule. In schematics on the right, blue represents DNA, yellow represents biotin-streptavidin, and red represents the cohesin holocomplex. (**B**) Snapshots and schematics of side-flow experiment before and after addition of cohesin complexes. Yellow arrow indicates a DNA region that is tightly clustered (i.e., not a DNA loop). (**C**) DNA cluster size as a function of time (right) calculated from the integrated fluorescence intensities in the cluster and the full 48.5-kbp λDNA in the image (left). Yellow circle indicates the compaction spot. (**D**) DNA compaction time under various conditions (*n* = 33, 25, 10, 13, 10, 13, 7, 11, and 9, respectively). (**E**) Cluster formation with labeled cohesin holocomplex showing the colocalization of compacted DNA (blue) and cohesin (red). (**F**) Representative measured trace of DNA compaction. (**G**) Simultaneously measured cohesin binding trace. (**H**) Histogram of bleaching step intensities of single Alexa647-cohesin molecules (*n* = 64). Inset shows a representative bleaching trace of a small cluster. A Gaussian fit (blue) yielded 3.0 ± 1.2 arbitrary units (a.u.) (means ± SD). (**I**) Number of cohesin holocomplexes in a cluster. A Gaussian fit (blue) yielded 740 ± 500 (means ± SD). (**J** to **L**) Atomic force microscopy (AFM) images of DNA/cohesin-holocomplex, cohesin-holocomplex only, and DNA only, respectively. (**M**) Volumes of the DNA/cohesin clusters for different conditions (median ± SEM; *n* = 21, 11, and 57). Red line in inset illustrates a cluster with its boundary (red). Two-paired Student’s *t* test was used for (D) and (M).

First, we visualized cluster formation by the *Saccharomyces cerevisiae* cohesin complex on DNA in vitro in real time (Fig. 1A and movie S1). We immobilized SYTOX Orange (SxO)–labeled double-tethered λDNA [48.5 kilo–base pairs (kbp)] on a polyethylene glycol (PEG)–coated surface and applied 10 nM cohesin holocomplexes (i.e., the cohesin tetramer Smc1-Smc3-Scc1-Scc3 and the cohesin loader Scc2-Scc4). Note that these cohesin holocomplexes are proficient in cohesin loader–stimulated ATP hydrolysis and topological loading onto DNA (fig. S1, A and B) (*7*, *24*). We tested these yeast cohesin holocomplexes extensively for their putative loop-extrusion activity, but we failed to observe any DNA-loop-extrusion activity over a very wide range of parameters and combinations of cohesin subunits. Instead, we observed the spontaneous accumulation of DNA spots along DNA molecules (Fig. 1A and movie S1). Application of an in-plane side flow (*5*) showed that these were stably condensed clusters (Fig. 1B and movie S2) and not DNA loops.

We observed clusters formed by cohesin holocomplexes both in the absence and presence of ATP, showing that this behavior is ATP independent. To quantify the kinetics of cluster formation, we measured the fluorescence intensity of the cluster region (see Materials and Methods) (*5*). Upon flushing in cohesin, the intensity at the cluster spot increased approximately linearly over time (Fig. 1C). After a compaction time of about 30 s, a plateau was reached, where the cluster comprised a sizeable amount of DNA (20 ± 8 kbp; for more examples, see fig. S1, C to E). Clustering proceeded slightly slower (~27 s) at room temperature than at 32°C (~14 s). We also tested whether the cohesin loader alone could induce cluster formation but found that it cannot: Here, instead of Scc2-Scc4, we used a stable N-terminal truncated version of Scc2 (named Scc2C) that is proficient in all previously known in vitro functions (*24*) and observed that the combination of the cohesin tetramer and Scc2C induced DNA cluster formation, whereas Scc2C only or cohesin tetramer only did not (Fig. 1D and fig. S1F). We conclude that the observed cluster formation is induced by ATP-independent interactions between the cohesin holocomplex and DNA.

### Cohesin-DNA clusters contain many cohesin holocomplexes

The clusters contained a large number of cohesin complexes under these in vitro conditions, which we quantified by coimaging Alexa647-labeled cohesin complexes and SxO-labeled DNA. Cohesin complexes were observed to colocalize with the DNA clusters (Fig. 1E and movie S3), while hardly any cohesin was observed at other locations on the DNA. We consistently observed a simultaneous increase in both DNA (Fig. 1F) and cohesin intensity (Fig. 1G) in the clusters. To count the number of cohesin holocomplexes on the DNA, we compared the cohesin intensities of each cluster (Fig. 1I, top axis) with the intensity of single cohesin holocomplexes as deduced from bleaching steps in traces (Fig. 1H), yielding an estimate of 720 ± 470 (means ± SD) cohesin holocomplexes within a cluster (Fig. 1I, bottom axis).

Next, we visualized the clusters at higher resolution using atomic force microscopy (AFM) imaging of mixtures of λDNA (4 ng/μl) and 10 nM cohesin holocomplexes. Again, large DNA/cohesin-holocomplex clusters were observed when both cohesin and DNA were present (Fig. 1, J and M), while no cluster formation was observed for cohesin holocomplex only or DNA only (Fig. 1, K and L). The AFM images showed clusters with a dense protein-rich center and an outer region made of loosely compacted loops. These clusters showed clear cis-DNA clustering at these low DNA and cohesin concentrations, and hence, individual DNA blobs were well separated (~200-μm mutual distance in bulk solution; Fig. 1J). These DNA/cohesin-holocomplex clusters contained many cohesin holocomplexes in a broad distribution with a median value of about 170, a number that was estimated by dividing the average cluster volume by the volume of an individual cohesin holocomplex (Fig. 1M and fig. S2, A and B). Both the fluorescence and AFM data thus indicate a very large number of cohesin holocomplexes per cluster (where the lower estimate from AFM likely originates from the lower protein/DNA ratio). These results show that cluster formation is not due to DNA-independent oligomerization of cohesin holocomplexes but instead relies on interactions between cohesin holocomplexes and DNA.

### Cohesin-DNA clusters exhibit liquid-like behavior

**Fig. 2:**
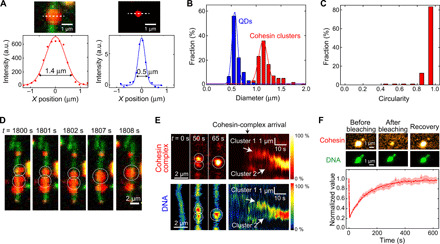
Cohesin holocomplex forms liquid droplets along DNA. (**A**) Images of a DNA/cohesin-holocomplex droplet (top left) and 20-nm QD (top right). White dashed lines indicate where cross-sectional intensity profiles were acquired, yielding the bottom panels with Gaussian fits. (**B**) Diameter distributions of cohesin droplets (means ± SD, 1.14 ± 0.18 μm; *n* = 151) and QDs (means ± SD, 0.57 ± 0.11 μm; *n* = 59). (**C**) Circularity distributions of cohesin droplets (*n* = 57). (**D**) Cohesin holocomplex (red) forms liquid-phase droplets along a DNA (green). Over time, two droplets are seen to fuse into one spherical droplet (movie S4). (**E**) Merging of two clusters, as monitored in the cohesin (top) and DNA (bottom) channels. Left shows three snapshots; right shows fluorescence intensity kymographs (*n* = 18). (**F**) Fluorescence recovery after photobleaching (FRAP) experiment of cohesin droplet. Snapshot images of a cohesin droplet on DNA, before and after photobleaching and after recovery (top). Cohesin cluster intensity versus time (bottom; mean ± SEM, *n* = 44). Time constant is 126 ± 4 s (error is SD).

Notably, the cohesin-DNA clusters displayed the behavior of liquid droplets. The clusters exhibited an average size of 1.14 ± 0.18 μm that exceeded the size of diffraction-limited spots measured for 20-nm quantum dots (QDs) (0.57 ± 0.11 μm) (Fig. 2, A and B, and fig. S3). The droplets were spherical in shape, as was quantitatively estimated (see Fig. 2C and fig. S3C), i.e., they exhibited a surface tension as expected for liquid droplets. When multiple clusters formed along a single DNA molecule (fig. S4, A and B), we often observed that two spherical neighboring clusters merged over time, where, subsequently, the shape of the resulting cluster again became spherical (Fig. 2, D and E, and movie S4). These features can be viewed as a defining behavior of liquid droplets and direct support for a type of phase condensation.

To monitor dynamic cohesin turnover within droplets, we turned to fluorescence recovery after photobleaching (FRAP) experiments. We bleached fluorescently labeled cohesin complexes in a droplet on a surface-tethered DNA and subsequently observed fluorescence recovery (Fig. 2F). The SxO-stained DNA intensity served as an internal control during these FRAP experiments as it stayed constant during the FRAP (Fig. 2F), confirming that the integrity of the cluster was maintained, while cohesin was bleached and exchanged with the environment during recovery. We observed a quick recovery of fluorescent cohesin after bleaching (τ = 126 ± 4 s from a fit to Fig. 2F). These data demonstrate the dynamic exchange of cohesin between the droplet and the environment (*25*), a characteristic feature of liquid-like clusters.

**Fig. 3:**
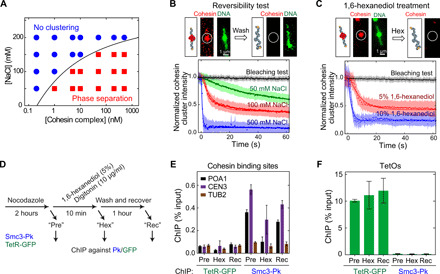
Reversibility of the cohesin-DNA droplet formation. (**A**) Phase diagram of cluster formation induced by cohesin holocomplex and λDNA at various salt (NaCl) and cohesin-holocomplex concentrations. Red squares indicate that DNA compaction occurred, while blue circles indicate that this did not occur. Solid line is a guide to the eye. (**B**) Reversibility test with washing in a high-salt buffer. Cohesin cluster intensities versus time upon washing with buffers with different salt concentrations (bottom; errors are SD). Dissociation times (τ) are 476 ± 25 s, 41.9 ± 0.7 s, 7.5 ± 0.2 s, and 0.79 ± 0.03 s (errors are SD) for bleaching, 50, 100, and 500 mM NaCl, respectively (*n* = 60, 113, 93, and 150). (**C**) Dissolving of a cohesin cluster by washing with 1,6-hexanediol. Cohesin-cluster intensity versus time upon washing with 1,6-hexanediol (bottom). τ = 476 ± 25 s, 7.9 ± 0.1 s, and 2.2 ± 0.1 s for bleaching, 5 and 10% 1,6-hexanediol buffer, respectively (*n* = 60, 80, and 37). (**D**) Scheme for in vivo 1,6-hexanediol treatment of yeast cells. (**E**) Chromatin immunoprecipitation–quantitative real-time polymerase chain reaction (ChIP-qPCR) results for cohesin (Smc3-Pk) and binding to a centromere (*CEN3*), chromosome arm (*POA1*), and a negative-control binding site (*TUB2*), before (Pre) and after 1,6-hexanediol (Hex) treatment and after washing out 1,6-hexanediol (Rec) (means ± SEM, *n* = 3). Tetracyclin repressor–green fluorescent protein (TetR-GFP) binding was analyzed as a control. (**F**) Same for binding to tetracyclin operator (TetO) sites (means ± SEM, *n* = 3).

Another typical feature of phase condensates is the reversibility of their formation. Cohesin-DNA clusters could be dissolved upon depleting the cohesin-holocomplex concentration in the buffer, or by increasing its salt concentration, thereby demonstrating the reversibility of the biomolecular condensation. A phase diagram of cluster formation shows that clustering is favored by low-salt and high-cohesin concentrations (Fig. 3A and fig. S4C), a feature observed for many phase-separating proteins (*10*). Cluster formation was observed at physiologically relevant concentrations of salt (~150 mM NaCl) and cohesin [>1 μM in yeast (*18*) and 333 nM in human (*19*)], suggesting that cluster formation may also occur in vivo. When the cohesin holocomplex was depleted by washing the channel with buffer, we observed the dissociation of the cohesin-DNA clusters as a decrease in the intensities of the cohesin holocomplex droplets on DNA (Fig. 3B). This implies that the clusters are dynamic, i.e., cohesins in clusters exchange with the pool in the bulk solution. The dissociation of cohesin holocomplex clusters was fastest at elevated salt concentrations, indicating that electrostatic interactions underlie droplet formation (Fig. 3B, bottom). As a further confirmation of the dynamic nature of cohesin clusters, we observed recovery of high salt–depleted DNA-cohesin droplets after subsequent readdition of cohesin holocomplexes (fig. S4D).

### 1,6-hexanediol disrupts cohesin-DNA clusters both in vitro and in vivo

Next, we used 1,6-hexanediol, an aliphatic alcohol that interferes with weak protein-protein (*26*) and protein–nucleic acid interactions (*27*) and is often used to differentiate liquid-phase and solid-like biological condensates as it dissolves liquid droplets but not gel-phase assemblies (*10*, *26*, *28*). In vitro, we observed a near-immediate disruption of cohesin-DNA droplets with 1,6-hexanediol treatment (τ = 2.2 s; Fig. 3C), which occurred faster than in typical other biomolecular condensation studies (*29*). Subsequent addition of cohesin holocomplexes in a buffer without hexanediol led to recovery of DNA-cohesin droplets (fig. S4E). Furthermore, we transiently applied 1,6-hexanediol to live yeast cells (Fig. 3D). Cohesin levels on chromosomes were monitored by chromatin immunoprecipitation (ChIP), followed by quantitative real-time polymerase chain reaction (qPCR). Ten minutes after administering 1,6-hexanediol, the amount of cohesin-associated DNA had noticeably decreased, which recovered again following 1,6-hexanediol washout (Fig. 3E).

This suggests that a portion of cellular cohesin reversibly associates with chromosomes through weak macromolecular interactions, consistent with phase separation. Notably, some cohesin remains bound to the DNA even in the presence of 1,6-hexanediol, suggesting a fraction of cohesin complexes that remains stably bound to DNA, which is likely topologically loaded cohesin that is resistant to harsh chemical treatment (Fig. 3E) (*24*, *30*). To test whether 1,6-hexanediol might have nonspecifically disrupted DNA-protein interactions, we monitored the association of a tetracyclin repressor–green fluorescent protein fusion protein with tetracyclin operators contained in the same strain. Its chromatin binding remained unaltered upon 1,6-hexanediol treatment (Fig. 3F), indicating that DNA binding of cohesin in vivo is uniquely susceptible to an agent that disrupts weak macromolecular interactions.

### Cohesin-DNA cluster formation is critically dependent on DNA length

**Fig. 4:**
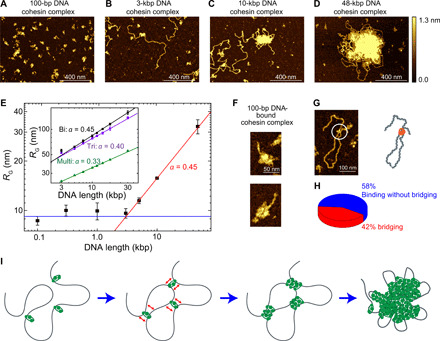
AFM imaging of DNA-mediated cohesin clusters and BIPS model. (**A** to **D**) Representative AFM images of cohesin holocomplex as it binds to different DNA lengths of 100 bp, 3 kbp, 5 kbp, and 48.5 kbp, respectively. (**E**) Radius of gyration of cohesin-DNA clusters versus DNA length (median ± SEM). Note the log-log scale. At low DNA length *l*, the cluster size is constant (green line), while beyond a critical length *l*_C_ ≈ 3 kbp, the data exhibit a power law, *R*_G_~*l*^α^ (blue line) with α = 0.45 ± 0.01 (SD) from a fit. Inset shows data from molecular dynamics (MD) simulations for cohesin with biconnectivity (black, α = 0.45 ± 0.01), triconnectivity (violet, α = 0.40 ± 0.01), and multiconnectivity (green, α = 0.33 ± 0.01). (**F**) Examples of single cohesin holocomplex bound to 100-bp DNA. (**G**) Representative image of DNA bridging by a single cohesin holocomplex. (**H**) Probability that a single cohesin holocomplex that bound DNA did or did not bridge to another segment along the DNA (*n* = 81). (**I**) Working BIPS model for cohesin-mediated phase separation. Bare DNA is bridged by a single cohesin holocomplex, increasing the local concentration of DNA, which subsequently induces binding of more cohesin holocomplexes to this region, leading to the formation of a large DNA/cohesin-holocomplex droplet. The cartoons are not drawn to scale.

Unexpectedly, the cohesin-DNA cluster formation critically depended on DNA length (Fig. 4, A to F). For short DNA lengths *l*, the cluster size, characterized by its radius of gyration *R*_G_, remained insensitive to *l* (Fig. 4E, blue line), whereas beyond a critical value of *l*_C_ ≈ 3 kbp, we observed that the cluster size strongly increased with DNA length, scaling as a power law *R*_G_~*l*^α^, with α= 0.45 ± 0.01 (Fig. 4E, red line; error is SD). The length-independent cluster size at short DNA length can simply be attributed to the size of single cohesin-holocomplex binding to DNA (Fig. 4, A and F). The absence of any clustering for short DNA indicates that cohesin binding to DNA does not simply trigger cohesin-cohesin interaction, e.g., through some conformational change. The critical value *l*_C_ ≈ 3 kbp that marks the onset of notable length-dependent clustering is in remarkable agreement with the length of DNA for which thermal fluctuations can induce spontaneous looping, i.e., *l*_C_ = 2π^2^*l*_P_≅ 987 nm ≅ 2903 bp for a persistence length *l*_P_ = 50 nm (see the Supplementary Materials). These stochastic thermal loops can be stabilized by a cohesin holocomplex that subsequently acts as a nucleation point for cluster formation (*21*, *22*), whereupon further clustering is entropically and energetically favored over a dispersed cohesin distribution. The 3-kbp critical length that is observed is the minimal length scale where bare double-stranded DNA is able to spontaneously (i.e., merely driven by thermal fluctuations) bend back to itself. For shorter lengths, DNA is simply too stiff to reach back to itself by thermal fluctuations, and hence, no clusters can be nucleated.

The power-law scaling of cluster size with DNA length reveals underlying properties of the condensation. While a power-law scaling with α = 1/3 is generally associated with collapsed globular polymer conformations, we here find a higher exponent of α = 0.45, closer to that of an ideal polymer (α = 0.5) (*31*). We performed molecular dynamics (MD) simulations in which DNA binding bridges are modeled as patchy particles with 2, 3, or many (~10) binding sites (see Fig. 4E, inset; fig. S5, A to E; and movie S5). As expected, we found that the many-binding case led to the formation of a compacted globule (fig. S5C) with α = 0.33. By contrast, clusters formed by cohesin bridges with *n* = 2 or 3 binding sites induced a qualitatively different condensation with exponents of 0.45 and 0.40, respectively (Fig. 4E, inset, and fig. S5D), with the *n =* 2 result in excellent agreement with our AFM data. The MD simulations for lower *n* showed cohesin-rich droplets that were rather porous and penetrable to diffusing solutes of sufficiently small size. Notably, while cohesin models with many (~10) binding points induced, as expected (*22*), Hi-C checker-board patterns that are qualitatively similar to mammalian compartments (fig. S5, F and G) (*11*) bridges with only two or three binding sites induced only very weak long-range compartments in in silico Hi-C maps, in line with experimental data for budding yeast cohesin that show only weak compartmentalization (*32*, *33*).

### Bridging-induced phase separation explains the cohesin-DNA clustering

Our results suggest that a bridging-induced phase separation (BIPS) model (Fig. 4I) underlies DNA-mediated cohesin clustering. In such a scenario, formation of droplets is initiated via cohesin holocomplexes that bridge the DNA polymer (*23*, *34*), whereupon additional proteins bind near the first bridging sites, in turn yielding larger clusters (*21*). This process is driven by a positive feedback between the bridging and the local DNA concentration (*22*). Theory suggests that the feedback is active as soon as the proteins bind stably to DNA, and for reasonable values of protein-DNA interactions strength [3 to 5 *k*_B_*T* (where *k*_B_ is Boltzmann constant and *T* is the absolute temperature)], it is predicted to occur down to 10 nM protein concentration (*22*), which is much smaller than the concentration of cohesin in yeast (*18*).

Local bridging of distant segments along a DNA molecule is an essential element in BIPS. Using AFM imaging, we observed that single cohesin holocomplexes can bridge the DNA, implying (at least) two DNA binding sites within the complex, consistent with previous suggestions of multiple potential binding sites in yeast cohesin (*35*). Figure 4G is an example where a single cohesin holocomplex is seen to bridge DNA (see fig. S5H for more examples), which was obtained by incubating 3-kbp DNA and cohesin holocomplexes for a very short time. Here, more than 40% of single cohesin complexes that bound to DNA showed DNA bridge formation (Fig. 4H).

## DISCUSSION

Summing up, for a broad range of parameters, cohesin and DNA were found to separate into a phase of locally dense clusters surrounded by a more dilute phase. We identified this ATP-independent cohesin-DNA cluster formation as a type of a phase separation. The liquid-like clustering behavior was found to be strongly DNA length dependent, indicative of bridging induced-phase separation that uses DNA-cohesin-DNA bridges as nucleation points for recruiting further cohesin complexes. Our data thus support a class of phase separation (BIPS) that differs qualitatively from the common LLPS type that is driven by protein-protein interactions and that has been extensively reported. Notably, the AFM results showed that “cohesin only” samples did not show any clustering (Fig. 1K), indicating that the clustering was not induced by weak cohesin-cohesin interactions. Instead, the DNA bridging is an essential element in this new form of phase condensation as clusters only appeared beyond a critical DNA length of 3 kbp. This is a hallmark of BIPS that is absent in other forms of phase separation.

The BIPS clustering that we report here is a previously underappreciated form of protein-DNA phase separation. While, because of the finite size of the simulations (*21*), it was referred to as clustering in the original paper that introduced the concept, our experiments show that cohesin complexes separate into two phases: a low-density unbound pool in the bulk and locally denser clusters that exhibit a surface tension and whose components dynamically exchange with the pool. This separation is the defining hallmark of classic liquid-gas or liquid-liquid (depending on the behavior of the low-density phase) phase separation (*36*). On the basis of our calculations (see the Supplementary Materials) (*22*), the minimal DNA length needed to trigger this phase separation of cohesin into a low-density pool and locally denser clusters is a free-energy minimizing solution of the system composed by DNA and cohesin proteins, well in line with classic thermodynamics of phase separation (*37*). Furthermore, our findings suggest that the phase separation occurs via nucleation rather than spinodal decomposition, as instead observed in some optogenetically activated proteins (*38*).

Cluster formation by cohesin holocomplexes in our experiments depended on inclusion of the Scc2 cohesin loader. In vivo, cohesin holocomplexes alternatively include either the cohesin loader or the related HEAT repeat containing cohesin subunit Pds5. Similar to the cohesin loader, Pds5 is known to be able to engage directly with DNA (*39*). The relative distributions of cohesin loader and Pds5-containing complexes and their interconversion are incompletely understood. A further investigation of Pds5-containing cohesin complexes and their possible DNA-dependent clustering behavior will be important questions for future investigations.

In budding yeast cells, a fraction of cohesin has been observed to remain associated with chromosomes in an Scc2-dependent manner even following DNA replication, especially in the vicinity of centromeres (*40*, *41*). Furthermore, investigations of cohesin bound to DNA in human cells found two subpopulations that are associated with chromosomes with different stabilities (*42*). All these observations are consistent with the possibility that a part of cohesin binds to DNA by topological entrapment, while an additional portion is recruited via bridging-induced phase separation.

Our observations of cohesin phase separation may help to interpret previous unsolved questions about genome organization by SMC proteins. For example, SMC-mediated phase separation explains why many previous in vitro studies observed higher-order assemblies with DNA and SMC proteins in the absence of ATP (*13*–*16*). Furthermore, in vivo, yeast cohesin has been observed to exist in foci on spread chromosomes (*43*), where each focus might consist of 5 to 20 cohesin complexes (*44*). In addition, a recent superresolution microscopy study showed 5 to 15 cohesin complexes per cluster in live mouse embryonic stem cells (*45*). Pronounced (~100 nm) cohesin/CCCTC-binding factor (CTCF) clusters were observed by Photoactivated localization microscopy (PALM) in mice cells (*46*). Clusters might furthermore form in local cohesin enrichment regions such as centromeres or sites of transcriptionally silenced regions (*47*). Recent micro-C experiments also observed neighboring cohesin binding sites to be in close contact (*48*). All these observations of clustering are consistent with cohesin phase separation, where the restricted DNA access by other DNA binding proteins such as nucleosomes may limit cluster size (*49*). Interallelic complementation between two mutant alleles in the same cohesin subunit has provided functional evidence for as yet unexplained cohesin-cohesin interactions (*50*). Phase separation could explain these interactions and in vivo chromatin recruitment of cohesin holocomplexes that are unable to topologically embrace DNA on their own (*51*). In addition, phase separation induced by cohesin complexes may explain how DNA loops can be stabilized at CTCF-bound sites in vertebrates. As CTCF is unstably bound to DNA (residence time, 1 to 2 min) and cohesin is more stably bound (residence time, 22 min) (*46*), cohesin phase separation at the stem of loops could stabilize cohesin-CTCF complexes. Last, our simulations showed that cohesin phase separation only yielded weak long-range compartment patterns, which notably is consistent with experiments that showed stronger A/B compartmentalization upon cohesin depletion (fig. S5, F and G) (*11*).

In conclusion, the demonstration that cohesin is a protein that induces biomolecular condensation reveals a basic principle for organizing genome architecture that potentially may be a generic feature of other SMC proteins as well. The BIPS that we observe for SMCs on DNA expands the range of phase-separation phenomena, as it involves the polymeric nature of long DNA molecules as a key ingredient in phase separation. BIPS has great explanatory power for aspects of chromosome organization that will be interesting to explore further.

## MATERIALS AND METHODS

### Protein purification

Cohesin tetramer (Smc1, Smc3, Scc1, and Scc3), Scc2C, and cohesin loader (Scc2-Scc4) were purified by following protocols described in a previous paper (*24*, *52*). For the labeling of cohesin, integrative plasmids p*GAL-SMC1-Pk::ADE2*, p*GAL-OptSCC1-3C-ProtA::HIS3*, p*GAL-SMC3-SNAP::TRP1*, and p*GAL-SCC3-myc::URA3* were transformed (strain Y5345) to obtain SNAP-fused Smc3 at the C terminus. For this complex, we used the same purification protocol.

### Fluorescent labeling of cohesin

We mixed 1.16 μM SNAP-tag cohesin tetramer with 40 μM SNAP-Surface Alexa Fluor 647 (New England Biolabs) in a 20 mM tris (pH 7.5), 150 mM NaCl, 0.05% (w/v) Tween 20, 3% (w/v) glycerol, bovine serum albumin (BSA; 0.1 mg/ml), and 1 mM dithiothreitol and incubated the mixtures overnight. Labeled protein was separated from free fluorophore using a Zeba Microspin 40 kDa (Thermo Fisher Scientific), we filtered free fluorophores in 20 mM tris, 150 mM NaCl, 0.05% Tween 20, and 3% glycerol.

### Preparation of biotin-labeled λDNA

λDNA was labeled with biotin at both ends following protocols in a previous paper (*5*).

### ATP hydrolysis assay

A high-throughput colorimetric adenosine triphosphatase (ATPase) assay (PiColorLock, Expedeon) was used to measure the ATPase activity of cohesin holocomplex by following the manufacturer’s protocol. ATPase reactions were set up in total volumes of 20 μl containing 40 mM tris (pH 7.5), 25 mM NaCl, 2.5 mM MgCl_2_, 2.5 mM ATP, and 0.5 mM tris(2-carboxyethyl)phosphine (TCEP). Concentrations of λDNA (100 ng/μl; Promega) and 50 nM cohesin with or without 50 nM loader were used. Reactions were initiated by the addition of cohesin holocomplex and incubated for 15 min. Temperature was controlled using a PCR machine. ATP hydrolysis was halted by adding 5 μl of PiColorLock reagent into the reaction mixture, which also initiated color development. After 2 min, a stabilizer was added and thoroughly mixed to stop the coloration. Using a NanoDrop spectrophotometer, the absorbance was measured at 640 nm. The hydrolysis rate was calculated by dividing the total amount of phosphate that was produced by the total reaction time.

### Single-molecule fluorescence assay

Microfluidic flow chambers for fluorescence imaging were prepared by following an established protocol (*53*). Chamber dimensions were 3 mm by 15 mm by 100 μm. Piranha was used to clean a quartz slide to which 12 holes were drilled. The quartz slide and cover slips were PEGylated with a 1:100 ratio of biotin-PEG and PEG by dissolving the powders into sodium bicarbonate solution (0.1 M NaHCO_3_, pH 8.5). After washing and drying the PEGylated slides, the flow chambers were assembled using double-sticky tape that defined six chambers, and the edges of the chambers were sealed by epoxy glue. Polytetrafluoroethylene tubing was connected to the drilled holes on one side of each chamber, and a reservoir was built using a pipette tip.

Buffer flow was controlled with an electric syringe pump. First, T50 buffer [20 mM tris (pH 7.5) and 50 mM NaCl] was injected into the flow channel. To tether the DNA onto the PEG surface, streptavidin solution (100 μg/ml) was flushed for 30 s for streptavidin to bind to the biotin-PEG, followed by washing with T50 buffer. A solution of 48.5-kbp double-biotinylated λDNA (100 pg/μl) in T50 buffer was injected at an initial flow speed of 20 μl/min for 30 s. After that, a reduced flow speed of 3 μl/min was maintained for 20 min. Unless stated otherwise, single-molecule fluorescence cohesin studies were performed in a reaction buffer of 50 mM tris (pH 7.5), 50 mM NaCl, 2.5 MgCl_2_, 0.5 mM TCEP, BSA (0.5 mg/ml), 10 to 50 nM SxO, and 2.5 mM ATP. Most experiments were performed using 10 nM cohesin holocomplexes (cohesin tetramer and cohesin loader). Lower concentration of SxO was used to minimize labeling artifacts. To observe the phase diagram of Fig. 2C, we used a variety of NaCl and cohesin-holocomplex concentrations. For experiments with labeled cohesin, an imaging buffer was used of 100 mM tris (pH 7.5), 50 mM NaCl, 2.5 mM MgCl_2_, 50 nM SxO, and the oxygen-scavenging system [2 mM trolox, 1% glucose, glucose oxidase (300 μg/ml), and catalase (30 μg/ml)].

For imaging of the SxO-stained DNA only, a 561-nm laser excitation was used. For dual-color colocalization imaging of SxO-stained DNA and Alexa647-labeled cohesin, an alternating-laser excitation mode was used with 561- and 642-nm laser excitation, respectively. We used a custom-modified inverted Nikon epifluorescence microscope equipped with a Nikon 100×/1.49 Apo total internal reflection fluorescence oil immersion objective. Image acquisition started immediately after injection of the reaction buffer. Highly inclined and laminated optical sheet mode was used for imaging, and temperature was controlled (Okolab). Images were acquired by a charge-coupled device camera (Andor iXon Ultra 897) with a dual-emission image splitter (OptoSplit) for dual-color experiments. MetaMorph software was used to record the single-molecule fluorescence images.

### FRAP experiments

To induce cohesin-DNA droplet formation in the microfluidic chamber, we introduced 10 nM Alexa647-labeled cohesin and 30 nM cohesin loader in the reaction buffer (see above) without the oxygen-scavenging system. After the droplet formation saturated (~10 min), we bleached the droplet by exciting the laser with consecutive 20-ms exposure times without a time gap. To monitor recovery of the fluorescence, we imaged the droplets using 10-s time gaps between two consecutive frames with 20-ms exposure of the laser, to minimize photobleaching of the labeled cohesin complexes. The intensities were normalized to the intensities of the droplets without bleaching. Recovery time constant was obtained by a single-exponential fit.

### In vitro high-salt wash and 1,6-hexanediol treatment experiments

Cohesin-holocomplex droplets were formed on doubly tethered DNA molecules after 5 min injection of 10 nM Alexa647-cohesin holocomplex. Then, a different salt buffer [50 mM tris (pH 7.5), 50/100/500 mM NaCl, 2.5 mM MgCl_2_, 250 nM SxO, and the gloxy oxygen-scavenging buffer] was injected (Fig. 2, D and E). For the 1,6-hexanediol experiment, 5% (w/w) or 10% 1,6-hexanediol (Sigma-Aldrich) in a reaction buffer [50 mM tris (pH 7.5), 50 mM NaCl, 2.5 mM MgCl_2_, 250 nM SxO, and the oxygen-scavenging system] was injected. The decrease in the Alexa647-cohesin droplet intensities was monitored using a 642-nm laser (Fig. 2, E and G). Dissociation time constants were obtained by single-exponential fits.

### Image analysis and quantification

Immobilized λDNA fluorescence intensity profiles showing DNA clusters were obtained from the summation of the intensity values of 11 pixels obtained from a line perpendicular to the extended DNA in each frame. Background intensity was subtracted using a two-dimensional median smoothing. Afterward, to correct intensity fluctuations such as bleaching, the intensities were normalized by the maximum value during the measurements. The kymograph was constructed after obtaining the intensities for all frames the normalized intensity profiles.

For the kinetics analysis of DNA cluster formation, we defined the region of interest (ROI) of both the clustered area and the area of the entire DNA using ImageJ (Fig. 1C, left). Using ImageJ, we measured the sum of the intensities of the pixels in the ROI of each frame. Background intensities were subtracted. Using the intensity information, the size of DNA (in kilo–base pairs) in the clustered area was obtained by normalization with the sum of the entire DNA intensity and multiplication by 48.5 kbp (*5*). To obtain the compaction time, we smoothed the time trace using the Savitzky-Golay method with a moving window of 250 points. Then, we determine the compaction time between starting compaction point (5%) and the ending point (95%). To measure the kinetics of the cohesin cluster formation and release, we used the ROI where the Alexa647-cohesin holocomplex cluster was colocalized with the cluster formation, yielding intensity-time traces as shown in Fig. 1G. To measure the bleaching steps of a single Alexa647-cohesin holocomplex (Fig. 1H, inset), we did not use oxygen scavenging system (gloxy) to effectively bleach the Alexa647 fluorophores. To obtain the step size, we analyzed clusters whose initial intensity was comparably low, where only a limited number of steps (<8) were observed. We applied a step-finding algorithm following a previously described algorithm (*54*).

Cohesin droplet diameter and circularity were measured by Fiji and MATLAB (Fig. 2, A to C) (*10*). The cross-sectional intensity profiles of droplets were obtained by Fiji. The diameter was measured by the distance between two points at 20% of the maximum fluorescence intensity of the Gaussian-fitted graph of cross-sectional intensity profiles of a droplet and using a home-built MATLAB code (Fig. 2A). As a control, to show that our observed DNA/cohesin-holocomplex droplets are larger than diffraction limit, 20-nm QDs (Qdot 705, Thermo Fisher Scientific) were nonspecifically adsorbed on the slide glass and similarly analyzed. To extract the diameter of a single QD rather than QD clusters, we analyzed QD fluorescence spots that showed blinking events. To show that the droplet is spherical, the cluster shape was deduced using Fiji by applying a threshold of 20% intensity of the average maximum intensity of the Gaussian fits of the droplets (Fig. 2C) (*10*). Then, the circularity was measured as 4π*A*/*P*^2^, where *A* and *P* are the area and perimeter of the droplet, respectively, and it shows how closely the shape resembles a perfect circle with a circularity of 1.

### ChIP experiments using 1,6-hexanediol treatment of yeast cells

Cells were grown in yeast-peptone-dextrose medium to mid-exponential phase before addition of nocodazole (8 μg/ml) for 2 hours to achieve a mitotic arrest. A first aliquot of the culture was retrieved for ChIP analysis. 1,6-Hexanediol was added at a concentration of 5%, together with digitonin (10 μg/ml) to permeabilize the cell membrane for 10 min—conditions that did not impede cell growth or survival following washout. After taking a second aliquot, the culture was filtered, washed, and resuspended in fresh medium containing nocodazole, but no 1,6-hexanediol for recovery and the final aliquot was harvested after 1 hour. ChIP followed a previously published protocol (*35*). Briefly, cells were fixed with formaldehyde and harvested. Protein extracts were prepared and chromatin disrupted by sonication. DNA fragments cross-linked to the protein of interest were enriched by immunoprecipitation. After reversal of cross-links, DNA from both immunoprecipitates and whole-cell extract was purified and quantified using the PowerUp SYBR Green Master Mix (Thermo Fisher Scientific) and the ViiA 7 Real-Time PCR System (Thermo Fisher Scientific). The primer sequences used are listed in table 1.

### AFM imaging and image processing

To image cohesin/DNA clusters, we mixed 10 nM cohesin holocomplex and DNA (4 ng/μl) of various lengths [0.1, 0.3, 0.5, 1, 3, 5, and 10 kbp (Thermo Fisher Scientific) and 48.5 kbp (Promega)] in a reaction buffer [50 mM tris (pH 7.5), 50 mM NaCl, 2.5 mM MgCl_2_, and 2 mM TCEP] in an E-tube and incubated the mixture for 5 min (Figs. 1, J to M, and 4). In the case of Fig. 4H, to show single cohesin-holocomplex–mediated DNA bridging, we mixed 1 nM cohesin holocomplex and 3-kbp DNA (4 ng/μl) and incubated them for a very short time (10 s). The 3-kbp-long DNA was used to avoid large cluster formation. Afterward, the mixtures were deposited onto mica that was pretreated with polylysine (0.00001%) (*8*). After briefly washing the sample using 3 ml of Milli-Q water, the sample was dried using a nitrogen gun.

AFM measurements of the dried sample were performed on a MultiMode B. (Bruker), with a NanoScope V controller and NanoScope version 9.2 software. SCANASYST-AIR-HR cantilevers (Bruker; nominal stiffness and tip radius of 0.4 N/m and 2 nm, respectively) were used. The PeakForce Tapping mode was used with an 8-kHz oscillation frequency, and a peak force set point value less than 70 pN was used to minimize sample invasiveness caused by sample and tip interaction. For imaging the proteins and protein/DNA mixtures, the scan area of 10 μm by 10 μm with 5120 pixel by 5120 pixel was recorded at the scanning speed of 0.7 Hz. All measurements were performed at room temperature.

For image processing of dry AFM images, Gwyddion version 2.53 was used. First, background was subtracted, and transient noise was filtered. To ensure that only the empty surface was used for background subtraction, masking particles and subtracting (planar and/or line by line) background polynomials were used by excluding the masked regions. Horizontal scars, which occasionally occur due to feedback instabilities or protein sticking to the AFM tip, were removed. Afterward, plane background subtraction was applied. Last, the blind tip estimation was used to estimate the shape of the tip, and surface reconstruction was performed to reduce the broadened effects caused by tip convolution (the widening of images induced because the AFM tip size is not zero) (*55*).

To measure the volume of a single protein (fig. S2), using Gwyddion, we masked each protein on the images and performed grain volume measurement to obtain the volume information of the each masked protein. For the cluster volume measurements, the territory of the proteins areas bound to DNA was defined by Gwyddion manually. The volume of the defined area was obtained by Gwyddion. To analyze the volume of the DNA/cohesin-holocomplex clusters, to obtain higher than 99.9% confidence level, 7 SDs and the average of the volume of a single cohesin-holocomplex were used as a threshold (2134 nm^3^). The defined area whose cluster volume is higher than the threshold was considered as a cluster.

## SUPPLEMENTARY MATERIALS

Supplementary material for this article is available at http://advances.sciencemag.org/cgi/content/full/7/7/eabe5905/DC1

## Appendix

View/request a protocol for this paper from *Bio-protocol*.
